# Grand and Less Grand Challenges in Avian Physiology

**DOI:** 10.3389/fphys.2017.00222

**Published:** 2017-04-19

**Authors:** Colin G. Scanes

**Affiliations:** Center of Excellence in Poultry Science, University of ArkansasFayetteville, AR, USA

**Keywords:** challenge, reproduction, birds, poultry, gastro-intestine, stress

A series of grand challenges in avian physiology are made. These challenges include determining the following. How the metabolic demands of flight are met including during migration and the unique control mechanisms? What is the neural, endocrine and paracrine control of gastro-intestinal functioning? How is the reproductive system of the hen controlled including recruitment and hierarchy of follicles, circadian pattern of ovulation and oviposition, cross-talk between the ovary and oviduct and neural, endocrine and paracrine control of the functioning of the oviduct? What are the roles of avian cytokines in the control of immune functioning and if there are non-immune related actions? What are the physiological adaption mechanisms that allow unique life patterns in avian species such as living in very cold water or on ice for penguins? When a function such as flight is lost/gained multiple times, what are the impacts on physiological control mechanisms? How can the techniques such as proteomics, metabolomics and microbiomics be applied to understanding the physiology of birds? How can existing and novel avian models be applied in new ways to biomedical investigations? Once we have fuller understanding of the effects of stress in birds, the challenge will be to understand the physiology of an unstressed bird?

## Introduction

Avian physiology has two distinct branches; namely the physiology of wild birds (e.g., flight, migration and seasonal breeding) and the physiology of poultry (domesticated birds consisting of predominantly chickens but also domestic ducks, geese, turkeys and ratites—ostriches, emus, and rheas). This division is based in part on the concept that there have been marked shifts in the genetics of poultry species due to domestication followed by breeding over millennia in a domesticated state and then to scientific selective breeding programs for well over 60 generations. Some researchers studying wild birds consider that selection for growth and egg production makes the physiology of poultry unrepresentative of birds in general. In addition, there are organizational differences. Individuals in university departments of animal or poultry science make up a sizable group investigating the physiology of poultry. These researchers frequently attend professional meetings of the Poultry Science Association and branches of World's Poultry Science. Others attend the Society for the Study of Reproduction or the Society for the Study of Reproduction and Fertility or specific national meetings. In contrast, research on the physiology of wild birds is mainly carried out in University departments of biology, biological science, systems biology or ecology departments. Researchers attend a variety of different professional societies including ornithology societies (such as the American Ornithological Society) or physiological societies (e.g., American Physiological Society and Physiological Society). Unfortunately, there is a lack of interaction between the two research communities.

What are the grand challenges in avian physiology? There are at least three categories of grand challenges:

- Attitudinal challenges.
- Conceptual challenges.
- Research challenges.

## Attitudinal challenges

It might seem surprising to include attitudinal challenges in this narrative. However, scientists are people and as much as we strive for objective and unbiased research, there are challenges. Firstly, is what we understand truly representing the real situation? Researchers tend to avoid re-examination of the “known.” The “known” can represent misreading of original research or often-repeated fallacious conclusions of well-respected leaders in the field. New techniques allow complete re-examination of dogma and the possibility to refute falsehoods. Are the gaps well established? This will be returned to under the topic research challenges below? Another challenge is that the assumptions made may be erroneous. This might be analogized to trying to complete a jigsaw puzzle. Do the pieces fit? If yes, are they the right pieces? Are they in the right place? Does the puzzle have all the pieces? In biology, we never have all the pieces. Another challenge is that older work is often neglected or dismissed or even totally ignored. Human intellect and ingenuity has not changed in the last 10,000 years. Moreover, there was excellent science, 25 or 50 or 100 years ago.

## Conceptual challenges

To assume that work on one species can be simply overlaid on to another is fallacious. This is particularly problematic trying to overlay the physiology of laboratory rodents on to birds. This is also true irrespective of whether it is assuming that the physiology of a domestic duck and/or domestic turkey and/or chicken are the same or that the physiology of a wild bird is directly transposable to another species. Similarly, research on the physiology of one population of a wild bird species may not reflect the situation in other populations of the same species due to selection pressure for the particular locale. Similarly, the physiology of layer chickens selected for number of eggs produced is likely not be identical to that of broiler chickens selected to growth, feed efficiency and breast size. This is not to say that research on one species does not inform research on another. Research on poultry is relevant research on wild birds and *vice versa*. Indeed, this is the *raison d'etre* for this new section.

There are other multiple conceptual challenges to avian physiology. Examples of these conceptual challenges include incorporation of seemingly disparate fields into avian physiology. These include evolutionary biology, systematics and ecology, genomics, transcriptional genomics, proteomics, metabolomics, microbiomics, animal behavior, neuroscience, toxicology and pathology. Examples of these will be considered below.

It is argued that understanding the evolutionary, systematic and ecological background of a species is critically important to inform physiological research as can genomics. The assumption that “a bird” is “a bird” is “a bird” is wrong. The importance of incorporating an evolutionary/systematics/ecological viewpoint is clearly seen from distance between the ratites with loss of flight multiple times (Harshman et al., 2008) and between chickens and ducks from other wild birds with a divergence/last common ancestor existing about 85 million years ago (e.g., Brown et al., 2008; Claramunt and Cracraft, 2015).

Once a functioning gene is lost, it is very unlikely to be recovered (Delfino et al., 2010). The well-being of birds is maintained without the products of the lost gene as is the case for cortistatin or neuropeptide B (Delfino et al., 2010). Moreover, gene duplication is necessary but not sufficient for the acquisition of new functions. For instance, there has been duplication of the growth hormone gene in Passerine birds (Yuri et al., 2008; Arai and Iigo, 2010) but the physiological significance and selective advantage of this is not understood.

Undoubtedly, genomics and the related fields of transcriptional genomics, proteomics and metabolomics have brought about a revolution in biology. These approaches are being utilized in avian physiology to various degrees. Proteins can undergo post-translational modification to generate peptides and either the protein or resultant peptide can be chemically and hence functionally modified, for instance phosphorylated or sulfated or acylated. What is often not known is which product is the physiologically important and under what circumstance?

Birds have been invaluable models in study of animal behavior (e.g., reviewed Clayton and Emery, 2015; Furuse, 2015), seasonal breeding and interactions with the environment (reviewed e.g., Wingfield et al., 2016),neuroscience, for instance, bird song (Reviewed e.g., Bailey and Saldanha, 2015; Mello and Clayton, 2015; Mori and Wada, 2015), magnetoreception (reviewed Mouritsen, 2015) circadian rhythms and extra-retinal light reception (Bertolucci and Foà, 2004; Reviewed: Kumar, 1997).

Conceptual issues focusing on reproduction are the following. There is a markedly different ovarian and oviductal structures in birds than in mammals. There is a single ovary and single oviduct in chickens. One oviduct regresses under the influence of anti-mullerian hormone (AMH) with AMH also causing the regression of the Mullerian ducts in the male (Johnson et al., 2008). In addition, AMH is expressed in follicles (Johnson et al., 2008) but the significance is not fully elucidated.

The ovary of chicken consists of primordial follicles, small white follicles, small yellow follicles, about six large yellow pre-ovulatory follicles and several post-ovulatory follicles. The large follicles grow to maturity over about 6 days. When the mature follicle (F_1_) is ovulated, an additional follicle(s) are recruited to the hierarchy. Alan Johnson's laboratory has conducted a series of elegant studies defining the roles of hormones, neuropeptides and growth factors [luteinizing hormone (LH), follicle stimulating hormone (FSH), vasoactive intestinal peptide (VIP), epidermal growth factor receptor ligands (EGFRL), transforming growth factor β (TGF β) and bone morphogenetic proteins (BMP4/6)] on different sized follicles in the chicken ovary (e.g., Ocón-Grove et al., 2012; Kim and Johnson, 2016; reviewed Johnson, 2015). Recently, the role of epigenetic changes within the follicles has begun to be elucidated (Zhu et al., 2015). What is still not completely clear is how the follicles are recruited, how the follicular hierarchy is maintained, whether there is cross-talk between the endocrine and nervous system and if there is a role for the post-ovulatory follicle.

Almost 70 years ago, Rothchild and Fraps (1949) demonstrated that progesterone can stimulate the pre-ovulatory LH surge in chickens. The changes in plasma concentrations of progesterone and LH during the ovulation cycle were reported almost 25 years later (Furr et al., 1973; Wilson and Sharp, 1973; Lague et al., 1975). Interestingly, both peaked about 6 h before ovulation. This is consistent with LH increasing progesterone production and/or progesterone evoking the LH surge. Progesterone stimulation of LH release varies during the ovulation cycle with strong responses either 4 h after and 12 h before an ovulation but there was a smaller response 12–8 h before ovulation (Wilson and Sharp, 1973, 1975). More recently, a role for androgens has been implicated as the androgen antagonist blocks the increases in estradiol, progesterone and LH (Rangel et al., 2006). Injections of neither estradiol nor testosterone evoke pre-ovulatory surges (Wilson and Cunningham, 1980). So, what is testosterone doing and where is acting? However, we still do not have a comprehensive picture of the control of hypothalamic-hypophyseal-gonadal axis in the female.

Chickens lay eggs in a sequence or clutch followed by a missed day of oviposition. This is followed by resumption of egg laying and another clutch. A 4 egg clutch being represented as XXXX-XXXX-XXXX. The time of oviposition becomes progressively later during the clutch. The clutches of oviposition reflect a similar pattern for ovulation and hence of the ovulation-inducing LH surge. Over 50 years ago, a “critical period” or open period was postulated as to the times when ovulation can occur (Fraps, 1954, 1965). This open period follows a diurnal (Fraps, 1954, 1965) or circadian pattern. The 24-h periodicity of ovulation or LH surge is a circadian rhythm synchronizing the hen's ovulation cycle with environmental cues (e.g., photoperiod) (Fraps, 1954, 1965). Ovipositions, ovulations and, hence, the LH surge and “open period” are restricted to a period of 12 h (Wilson and Cunningham, 1981). If the time of night (scotophase) was advanced to occur 6–8 h after ovulation, there was a delay in the pre-ovulatory LH surge (Wilson et al., 1985). Studies on ahemeral cycles are consistent with a circadian rhythm for ovulation with a periodicity of about 26 h (Morris, 1973). Ahemeral cycles are light dark cycles (hours of light plus hours of dark) different from 24 h. Studies have been performed in turkeys under continuous lighting such that the circadian rhythm(s) were free-running. The interval between pre-ovulatory LH surges (periodicity) was 26 h in early egg producing turkey hens (Liu et al., 2002). In Magang geese, there is an interval of 48 h (2 days) between ovipositions (Qin et al., 2013). What is absent are comprehensive studies using today's techniques including the investigation the effects of ahemeral cycles of less than 24 h in modern laying hens. Corticosterone may be one factor signal coordinating the diurnal rhythm (Wilson and Cunningham, 1980). However, the physiological controls underlying of clutches and the timing of ovulation is still not well understood.

There is a relationship between the functioning of the oviduct and at least that of the largest pre-ovulatory follicle. The relationship between oviductal and ovarian functioning in the hen was first advanced over 60 years ago with Huston and Nalbandov (1953) demonstrating that ovulation is blocked if surgical thread is placed in the magnum. In chickens, turkeys and coturnix quail, ovulation of the subsequent follicle occurs after, but within 30 min of, oviposition of the previous ovum (Woodard and Mather, 1964). The duration of the time an egg spends in the oviduct appears to be affected by the light: dark cycle. This is supported by the following. Eggs from hens on a 27 h ahemeral cycle are larger than those on a conventional (24 h long) day or light-dark cycle (Morris, 1973). Unfortunately, the mechanism by which oviductal functioning, oviposition and the next ovulation has as yet not been clarified. Is it hormonal and/or nervous?

## Research challenges

In the last 75 years, there have been tremendous advances in our knowledge of avian physiology but much remained undiscovered. A series of grand challenges, albeit representative, are issued below:

**Grand challenge 1**: How are the metabolic demands of flight, in the short term, and during migration, in the long term being met? What are the unique control mechanisms?
**Grand challenge 2:** We know that the functioning of the gastro-intestinal tract can be affected by a series of peptides. What is not known is the overall control mechanism and whether the critically important peptides are of neural (peptidergic) or local (autocrine and paracrine) or endocrine origin. In addition, what is controlling the release of gastro-intestinal peptides (gut stretching and/or chemical stimuli from specific nutrients and/or from specific groups within the gut microbiome). In addition, what is the physiologically relevant “cross-talk” between gastro-intestinal peptides and central nervous neural functioning.
**Grand challenges 3: Reproduction in hens:**

3A. How are follicles recruited and how is the follicular hierarchy maintained?
3B. How is the circadian pattern of ovulation and oviposition achieved? Are there ovarian and oviductal oscillators? What is the cross-talk between the ovary and oviduct?
3C. What is the neural, endocrine and paracrine control of oviductal functioning and of oviposition including pituitary and local released mesotocin and/or arginine vasotocin and/or prostaglandins of the E series. The control of passage of the egg through the oviduct is only understood in what might be argued is at a fairly rudimentary manner.

**Grand challenge 3:** There are large numbers of avian cytokines identified. Their role in the control of immune functioning is beginning to be elucidated but greater depth understanding is needed. What is not clear is whether these avian cytokines have non-immune related actions. Moreover, the ability of the immune system to influence the functioning of the central nervous system and/or gastro-intestinal tracts and systems are not well established.
**Grand challenge 4:** What are the physiological adaption mechanisms that allow unique life patterns in avian species such as living in very cold water or on ice for penguins, consuming decomposing tissues from the carcasses of dead animals in scavenging birds such as vultures and living in disparate environmental with extremes of temperature, rainfall and atmospheric pressure?
**Grand challenge 5:** When a function is lost/gained multiple times, are the impacts on physiological control mechanisms the same due to convergent evolution or, if there is redundancy, are different mechanisms adopted? Alternatively, have entire new control systems developed?
**Grand challenge 6:** To understand the hormonal control of growth.
**Grand challenge 7:** This is to employ the techniques of proteomics (and immune-concentration/partial purification) to generate complete data on proteins, peptides and their post-translationally-modified forms in cells, inter-cellular spaces and plasma and ultimately gain an understanding of the physiology of these peptides and proteins under difference circumstances. Particular attention should be played to the gastro-intestinal tract and to regions of the brain including those responsible for bird song. Metabolomics, coupled with non-radioactive isotopes, offer tremendous scope for developing knowledge on the control of the metabolism of birds ranging from a fast-growing broiler chicken to birds migrating thousands of miles.
**Grand challenge 8:** To use non-radioactive isotopes and other techniques of metabolomics to provide comprehensive information and ultimately to fully understand of the physiological control of metabolism in birds. It is becoming increasing evident that the microbiome of an animal markedly influences its functioning.
**Grand challenge 9:** This is to develop a database of sufficient depth to understand the relationships between the microbiome and gastro-intestinal functioning.
**Grand challenge 10:** This is to build on the existence strong avian models and to develop new avian models.
**Grand challenge 11:** This consists of the following, having first determined the influence of stressors including disease and specific toxicant. The challenge is to understand what the physiology of an unstressed bird is.

The importance of stress, toxicology and pathology to avian physiology can be manifest in the following situations:

- Are we investigating a physiological meaningful situation or an artifact of an infectious disease or stress or exposure to a toxicant such as oil?

Irrespective of whether or not we are.

- Do we understand the effects of specific environmental stressors (e.g., low or high temperature) or infectious disease (pathophysiology) or stress or exposure to a toxicant on the physiology?

All aspects of avian physiology will be covered in this section of Frontiers in Physiology.

## Author contributions

The author confirms being the sole contributor of this work and approved it for publication.

### Conflict of interest statement

The author declares that the research was conducted in the absence of any commercial or financial relationships that could be construed as a potential conflict of interest.
